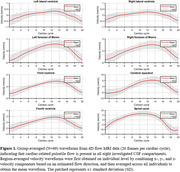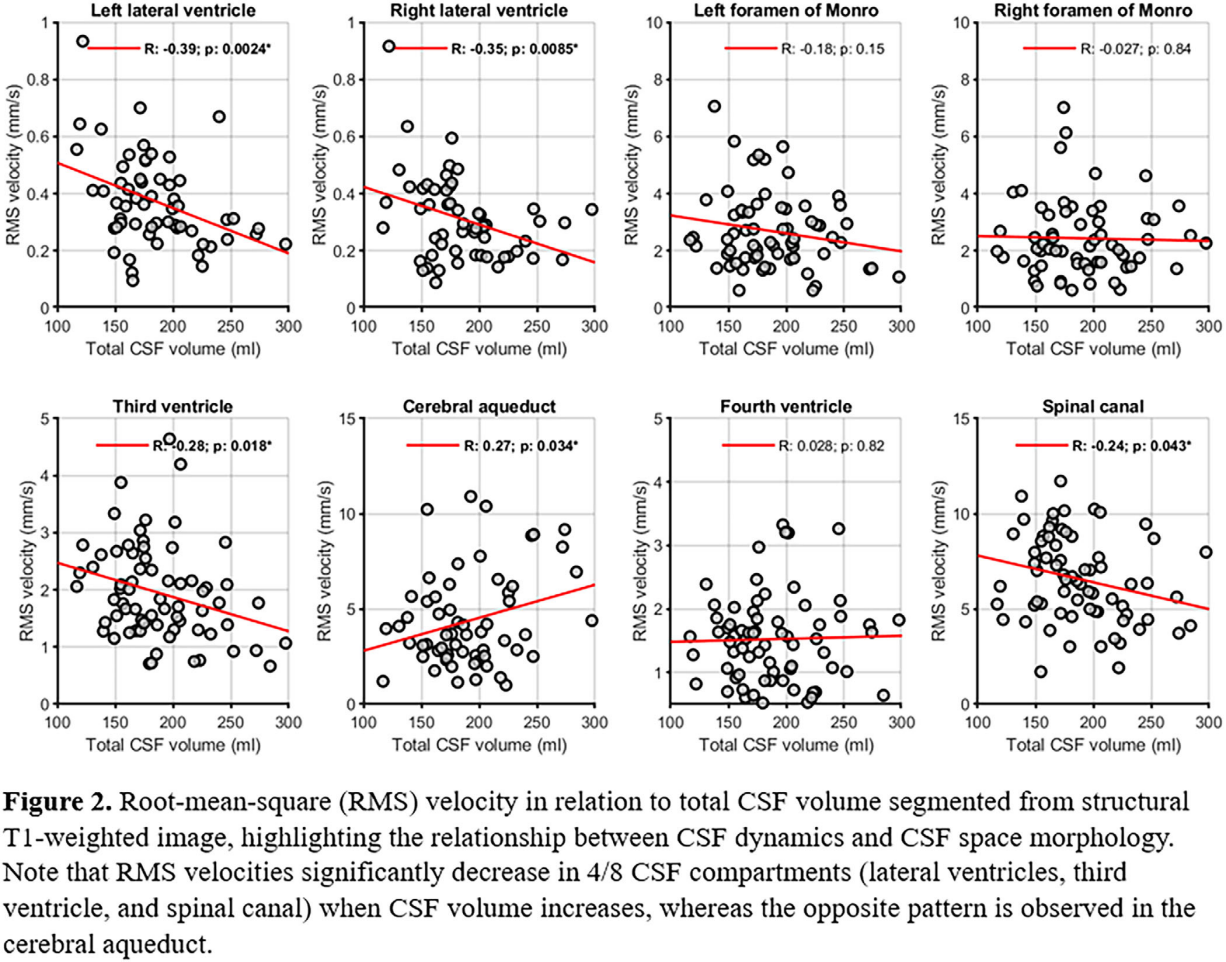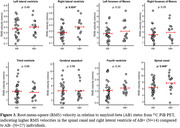# 4D flow MRI of CSF dynamics: relations to CSF morphology and Aβ status

**DOI:** 10.1002/alz.094049

**Published:** 2025-01-09

**Authors:** Tomas P. Vikner, Kevin M. Johnson, Sarah C. Hudson, Christian C. Pompa, Robert V. Cadman, William B. Bevis, Tobey J. Betthauser, Nathaniel A. Chin, Laura B. Eisenmenger, Sterling C. Johnson, Leonardo A. Rivera‐Rivera

**Affiliations:** ^1^ School of Medicine and Public Health, University of Wisconsin‐Madison, Madison, WI USA; ^2^ Department of Medicine, University of Wisconsin‐Madison School of Medicine and Public Health, Madison, WI USA; ^3^ Wisconsin Alzheimer's Disease Research Center, Madison, WI USA; ^4^ Alzheimer's Disease Research Center, University of Wisconsin‐Madison, Madison, WI USA

## Abstract

**Background:**

Cerebrospinal fluid (CSF) dynamics are increasingly studied to understand potential pathologic coupling with neurological disorders. In Alzheimer’s disease (AD), CSF dynamics may be altered secondary to AD‐related atrophy and enlarged CSF spaces. Additionally, animal studies suggest that altered CSF dynamics could impair clearance of metabolic waste, leading to accumulation of amyloid‐beta (Aß). However, assessment of human CSF dynamics has typically been limited to the cerebral aqueduct using 2D phase contrast MRI. In this work, we used 4D flow MRI to enable volumetric characterization of CSF flow dynamics in the human brain, to study its associations with CSF morphology and Aß burden from 11C‐PiB PET.

**Method:**

Cardiac‐resolved 4D flow MRI was used to assess CSF motion in N=69 participants (age 69±8 years; 17 Aß+) from the Wisconsin Registry for Alzheimer’s Prevention, using a clinical 3T scanner (GE Premier) and a velocity encoding of 5 cm/s. Region‐averaged velocity waveforms were acquired in the spinal canal (SC), fourth ventricle (4V), cerebral aqueduct (CA), third ventricle (3V), the foramens of Monro (FMo), and the lateral ventricles (LV), segmented from T1‐weighted volumes. The waveforms were characterized by root‐mean‐square (RMS) velocity and were evaluated in relation to total CSF volume and Aß status from an established PiB global distribution volume ratio threshold >1.16.

**Result:**

Velocity waveforms demonstrated cardiac‐pulsatility in all regions of interest (ROIs) (Figure 1), with RMS velocities ranging from high in the SC (6.56±2.29 mm/s) to low in the lateral ventricles (right LV 0.31±0.15 mm/s). RMS velocities were related to total CSF volume for the SC (R=‐0.24; p=0.043), CA (R=0.27; p=0.034), 3V (R=‐0.28; p=0.018), left LV (R=‐0.39; p=0.002), and right LV (R=‐0.35; p=0.009) (Figure 2). In preliminary analysis, RMS velocities were higher in Aß+ compared to Aß‐ for the SC (p=0.048) and right LV (p=0.024) (Figure 3).

**Conclusion:**

4D CSF flow MRI is feasible for the assessment of CSF dynamics throughout the ventricular system. Total CSF volume was related to RMS velocities in 5/8 ROIs, highlighting the influence of CSF morphology on CSF dynamics. Finally, Aß status was related to RMS velocity in 2/8 ROIs, indicating that altered CSF dynamics could play a role in incipient AD.